# Mitral Valve Fenestration as a Rare Cause of Congenital Mitral Insufficiency Successfully Repaired

**DOI:** 10.21470/1678-9741-2020-0262

**Published:** 2021

**Authors:** Francisco Fernandes Moreira Neto, Thiago S. Arantes, Mauro Cruz Jurca, Maria Rita F. S. Moreira, Cecilio A. Barbosa Jacob

**Affiliations:** 1 Clínica de Cirurgia Cardiovascular, Ribeirão Preto, São Paulo, Brazil.; 2 Department of Surgery, Faculdade de Medicina, Universidade de São Paulo, Ribeirão Preto, São Paulo, Brazil.; 3 Hospital São Lucas, Ribeirão Preto, São Paulo, Brazil.; 4 ProImagem Medicina Diagnóstica de Ribeirão Preto, São Paulo, Brazil.

**Keywords:** Mitral Valve Insufficiency, Tricuspid Valve, Heart Valve Diseases, Sutures, Pericardium

## Abstract

A rare case of congenital mitral insufficiency characterized by a fenestration in the anterior leaflet of mitral valve is reported. At operation, the mitral valve was successfully repaired by closure of unusual valvular tissue orifice with bovine pericardium and suture of the free edge between A1 and A2 without a ring annuloplasty.

**Table:** 

Abbreviations, acronyms & symbols	
**MR** **MV** **PDA**	**= Mitral regurgitation** **= Mitral valve** **= Patent ductus arteriosus**

## CASE PRESENTATION

The patient was born and then sent home the day after a standard cesarean delivery. He had his first regular pediatrician visit at 30 days of age and presented symptoms of shortness of breath. Due to quickly worsening symptoms, he returned to the emergency room when he was 2 months old, with severe dyspnea, hypotension, and had a cardiac arrest. After resuscitation maneuvers were successful, an emergency echocardiographic study revealed severe coarctation of the aorta, small patent ductus arteriosus (PDA), enlarged left atrium and ventricle, bicuspid aortic valve, and moderate mitral regurgitation (MR) (prolapsed anterior leaflet).

The prostaglandin was initiated, and the patient was brought to the operating room. The repair was done via left thoracotomy and, in addition to the coarctation and PDA, we also found an undiagnosed aberrant right subclavian artery originating in the descending aorta. Both were repaired. Against the odds, the MR persisted, even with almost absent aortic gradient and normal left ventricular function. Since the postoperative echocardiography failed to show an easily repairable lesion (now described as parachute valve), he was sent home with full treatment of diuretics, vasodilators, and digoxin.

**Fig. 1:**
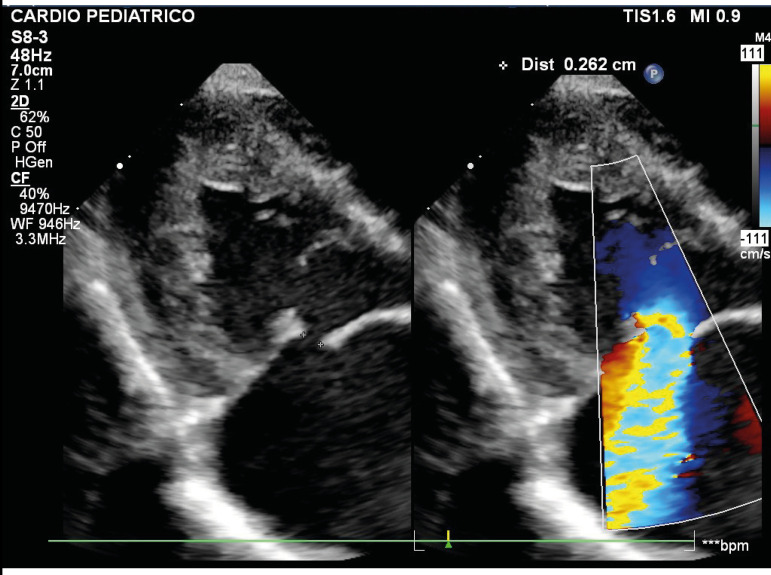
Cross-sectional echocardiography showing a perforation in the anterior leaflet of the mitral valve

In the follow-up, when he was 5 months old, he had persistent cardiac failure symptoms and was readmitted with New York Heart Association class IV. Marked cardiomegaly with the left atrium as the predominant chamber was seen on the chest radiograph associated with signs of pulmonary congestion. Electrocardiography showed sinus rhythm, right-axis deviation, and left atrium hypertrophy. The new cross-sectional echocardiography revealed a perforation in the anterior leaflet of the mitral valve (MV) (Figure 1 and Video 1) and the surgery was immediately recommended.

**Video 1:** Epicardial echocardiography in complement to view more details.

## TECHNICAL DESCRIPTION

Surgery was performed via a median sternotomy, using cardiopulmonary bypass, with moderate hypothermia (28°C) and cold potassium cardioplegia, and we could see a very enlarged left atrium. The MV was exposed directly via the left atrium and a 0.4-mm diameter gap like a hole on the anterior mitral leaflet was identified. The cordae were thick and an area of scar corresponding to the regurgitation jet was clearly visible (Video 2). The defect was closed with bovine pericardium patch in a running 6-0 polypropylene suture. Additional closure of the free edge between A1 and A2 was also done with 6-0 Prolene to achieve mitral competency and there was no need to perform annuloplasty or to use an annular ring. Saline solution was injected into the ventricular cavity through the valve and demonstrated a satisfactory repair with a good symmetric line of apposition between leaflets and almost absence of leaks. Furthermore, we opened the right atrium to close the patent foramen ovale and the final aspect can be seen in the Video 3. There were no difficulties coming off cardiopulmonary bypass. Cardiac output was good, and we left the operating room in need of only milrinone. He was extubated six hours after surgery, had an uneventful postoperative course in use of vasodilators, and the echocardiographic control reveal only small residual MR. He was discharged eight days after the operation only in need of captopril.

**Video 2:** Intraoperative view using cardiopulmonary bypass.

**Video 3:** Performing mitral valve repair and result.

## COMMENT

Mitral incompetence is a rare congenital malformation. It was first described by Semans and Taussig (1938). Primary congenital MV abnormalities are identified according to the four anatomic components of the valve ^[^^1^^]^. The most common lesion in congenital MV disturbance is an abnormality of the papillary muscles, followed by the involvement of leaflets, commissures, and chordae tendineae. This condition is often associated with anomalies involving the left side of the heart (especially mitral stenosis), or as part of a syndrome.

The etiology of congenital mitral insufficiency accompanying coarctation of the aorta is not clear. An embryologic association between the two lesions is difficult to explain, but it is possible for the same insult to cause unrelated congenital defects ^[^^2^^]^.

Despite advances in the treatment of complex congenital heart defects in very small infants, congenital mitral incompetence sufficiently severe to require an operation during the first year of life is still a therapeutic challenge. In view of its poor results, surgical treatment for congenital MV disease is always delayed until severe symptoms develop with medical treatment.

Contrary to the preoperative expectation, correction of the coarctation of the aorta and associated PDA before MV surgery did not result in improvement of cardiac failure. Removal of the aortic obstruction did not reduce the volume of blood regurgitating into the left atrium as we expected if the diagnostic of prolapsed leaflet was correct. Had the correct diagnosis been made, MR could have been corrected earlier, therefore avoiding the left atrium enlargement and all the complications in between the two operations.

## Conclusion

In reviewing the literature, we have found very few descriptions of patients treated surgically with congenital mitral incompetence and coarctation of the aorta. It is even more difficult to find an MR caused by a deficient tissue growth like a fenestration in the anterior leaflet, like this case ^[^^3^^]^. An understanding of the pathologic anatomy of congenital MR is absolutely relevant to its optimal surgical management. If one recognizes that regurgitation is due to a leaflet perforation, it may well be possible to patch the perforation, thereby avoiding MV replacement.

**Table:** 

Authors' roles & responsibilities	
FFMN	Substantial contributions to the conception or design of the work; or the acquisition, analysis or interpretation of data for the work; drafting the work or revising it critically for important intellectual content; final approval of the version to be published
TSA	Substantial contributions to the conception or design of the work; final approval of the version to be published
MCJ	Substantial contributions to the conception or design of the work; and acquisition of data for the work
MRFSM	Agreement to be accountable for all aspects of the work in ensuring that questions related to the accuracy of integrity of any part of the work are appropriately investigated and resolved; final approval of the version to be published
CABJ	Substantial contributions to the conception or design of the work; final approval of the version to be published
